# Mycorrhizal Fungi Isolated from Native Terrestrial Orchids from Region of La Araucanía, Southern Chile

**DOI:** 10.3390/microorganisms8081120

**Published:** 2020-07-25

**Authors:** Hector Herrera, Tedy Sanhueza, Rodolfo Martiarena, Rafael Valadares, Alejandra Fuentes, Cesar Arriagada

**Affiliations:** 1 Laboratorio de Biorremediación, Departamento de Ciencias Forestales, Facultad de Ciencias Agropecuarias y Forestales, Universidad de La Frontera, 4811230 Temuco, Chile; hector.herrera@ufrontera.cl (H.H.); t.sanhueza01@ufromail.cl (T.S.); alejandra.fuentes@ufrontera.cl (A.F.); 2 Estación Experimental Agropecuaria Montecarlo, Instituto Nacional de Tecnología Agropecuaria, Av. el Libertador 2472, Montecarlo N3384, Misiones, Argentina; martiarena.rodolfo@inta.gob.ar; 3 Instituto Tecnologico Vale, Rua Boaventura da Silva 955, Cep, 66050-090 Belém, PA, Brazil; rafael.borges.valadares@itv.org

**Keywords:** endophytes, mycoheterotrophy, mycorrhizal fungi, orchid mycorrhiza, orchids, symbiosis

## Abstract

Mycorrhizal interactions of orchids are influenced by several environmental conditions. Hence, knowledge of mycorrhizal fungi associated with orchids inhabiting different ecosystems is essential to designing recovery strategies for threatened species. This study analyzes the mycorrhizal associations of terrestrial orchids colonizing grassland and understory in native ecosystems of the region of La Araucanía in southern Chile. Mycorrhizal fungi were isolated from peloton-containing roots and identified based on the sequence of the ITS region. Their capacities for seed germination were also investigated. We detected *Tulasnella* spp. and *Ceratobasidium* spp. in the pelotons of the analyzed orchids. Additionally, we showed that some *Ceratobasidium* isolates effectively induce seed germination to differing degrees, unlike *Tulasnella* spp., which, in most cases, fail to achieve protocorm growth. This process may underline a critical step in the life cycle of *Tulasnella*-associated orchids, whereas the *Ceratobasidium*-associated orchids were less specific for fungi and were effectively germinated with mycorrhizal fungi isolated from adult roots.

## 1. Introduction

The symbiotic relationship established between orchids and their compatible mycorrhizal fungi is characterized by the dependence of the plant on fungal carbon (C) and other mineral nutrients essential to start the initial developmental stages [1,2,3]. These mechanisms are called mycoheterotrophy and describe the ability of the plant to obtain C from intracellular fungal hyphae [4]. Mycoheterotrophic processes are conserved in most orchids (accounting for roughly 30,000 species) with differing degrees of fungal exploitation, but are usually present at the seed germination stage, where an embryo without sufficient mineral nutrients to support morphogenesis needs the external supply of C from soil-borne fungi [5,6]. After the plantlet stage, orchids can retain mycoheterotrophy throughout their lives (fully mycoheterotrophic species), can change to autotrophy or can maintain both lifestyles (autotrophic and heterotrophic) in a process known as partial mycoheterotrophy or mixotrophy [4,7].

Mycorrhizal fungi associated with orchids are mainly included in the polyphyletic *Rhizoctonia*-like fungi complex, but recently the diversity of fungi establishing symbiotic interactions with orchids has grown considerably [8,9,10]. Additionally, differences in the fungi isolated from mycorrhizal roots can differ according to the root sections, sampling site, or plant stage (protocorm, plantlet or adult plant) [11,12,13]. A conserved characteristic is the formation of a symbiotic structure named peloton, which corresponds to hyphal coils where the metabolic interchange between symbionts occurs in both protocorm and mycorrhizal roots [1,14].

Chilean Orchidaceae are strictly terrestrial and require the nutrients obtained from the mycorrhizal fungi to germinate. After that, plants retain mycorrhizal structures in pelotons but photosynthetic tissues are also present. In the region of La Araucanía, almost 40% of the Chilean orchids are distributed in native and exotic forests, where it is expected that seeds have almost all of the symbiotic and nutritional requirements to germinate [15]. The soils in which these orchids grow are mainly of volcanic origin, with high organic matter content and high rates of nitrate and phosphate retention [16,17]. These conditions turn to orchid mycorrhizal fungi (OMF), as well as other microorganisms—essential symbionts—to achieve plant establishment and sustain growth when orchids defoliate or in winter where orchids live only by the reserves stored in their underground organs [8]. Culture-based methodologies have identified different *Ceratobasidium* spp. and *Tulasnella* spp. strains, as common OMF are associated with native terrestrial orchids [18,19,20]. These results showed that different OMF can be isolated from orchids from diverse geographic zones, as well as showing differences in their capabilities to induce seed germination under in vitro conditions [19,21,22].

Considering the dynamisms of the mycorrhizal associations of terrestrial orchids and the influence of the ecosystem conditions, we hypothesize that a narrow fungal diversity can be isolated from mycorrhizal tissues of native species from high-diversity microhabitats in the region of La Araucanía. Therefore, the aim of this work was to isolate and identify mycorrhizal fungi, able to promote the seed germination of terrestrial orchids, colonizing different microhabitats in the region of La Araucanía for their use in the germination of native threatened species.

## 2. Materials and Methods

### 2.1. Study Sites and Sampling

Terrestrial orchids colonizing several sampling sites, as shown in Table 1, were found on different field trips between November 2018 and February 2019 at various locations in the region of La Araucanía. We focused the sampling at sites with high orchid populations. These orchids were found growing in the first 15 cm of the soil in native grasslands and in the understory of native or exotic forests. Only colonized roots segment (brownish root) from random plants were extracted and transported to the laboratory for further processing.

During sampling, we identified 13 terrestrial orchid species with flowers, which was key to perform identification according to the flower characteristics detailed in Novoa et al. [15].

### 2.2. Isolation and Characterization of Fungi

Compatible mycorrhizal fungi were isolated from peloton-containing roots following the methodology proposed by Valadares et al. [23] with modifications. The sampled roots were washed under running tap water and inspected for the presence of mycorrhizal segments. The root segments containing pelotons were cut transversely in minor fragments (1 cm) and superficially disinfected by immersion in a solution containing 95% ethanol, sodium hypochlorite (2.5% active chlorine) and sterile deionized water (1:1:8), and were rinsed five times in sterile deionized water under a laminar flow cabinet. The disinfected root segments were sliced transversely and the pelotons were separated with a sterile scalpel, discarding the velamen and non-peloton-containing root segments. The fragments containing mycorrhizal fungi were placed in Petri dishes with modified potato dextrose agar (PDA, plus streptomycin at 100 mM) and cultured for 14 days in darkness at 25 ± 1 °C or until fungal growth started. The purified strains were cultured in PDA and grouped according to the isolation source, phenotypic characteristic of the strains and the growth rate. Isolates that did not match the standard phenotypical characteristics of OMF were discarded.

### 2.3. Molecular Identification and Phylogenetic Analyses

The fungal strains were identified according to Valadares et al. [23] with minor modifications. Fungal isolates (ø 5 mm mycelia plugs) were incubated in 50 mL falcon tubes containing 1/7 potato dextrose broth under continuous agitation in an orbital shaker at 150 rpm for 21 days. The fungal mycelia were washed with sterile deionized water and centrifuged at 5000 rpm. The pellet underwent DNA extraction to perform molecular identification based on the nucleotide sequence of the internal transcriber spacers (ITS). Total DNA was extracted with the DNeasy Plant Mini Kit (Qiagen, Hilden, Germany), according to the manufacturer’s instruction. The ITS region was amplified by using the ITS1 and ITS4 primers, according to White et al. [24]. The PCR cycle consists of initial denaturing at 95 °C for 5 min, followed by 30 cycles of denaturing at 95 °C for 1 min, annealing at 55 °C for 1 min, extension at 72 °C for 1 min each and final extension for 5 min at 72 °C. PCR products were checked in 2% agarose gel stained with GelRed^®^ (Biotium Inc, Fremont, CA, USA). Sequencing was performed by Macrogen (Seoul, South Korea) and the sequences were submitted to the GenBank database.

The phylogenetic analyses of the sequences were performed according to Herrera et al. [19] with modifications. BLAST searches were performed to find the closest match in the database, accepting the species when the similarity between query and match was >99%, and the genus was accepted when similarity >95%. Multiple sequence alignments were performed using ClustalX with default conditions for gap opening and gap extension penalty [25]. Non-conserved regions were eliminated using the BioEdit software. Operative taxonomic units (OTUs) were assigned at 97% sequence similarity. Phylogenetic trees were constructed in the MEGA 6 software with the neighbor-joining method [26] and considering the sequences of mycorrhizal fungi isolated from native Chilean orchids, downloaded from the GenBank database [8]. Isolates that matched with OMF strains were stored at −80 °C in cryotubes with 10% Glycerol solution for further analyses.

### 2.4. Symbiotic Seed Germination

Symbiotic seed germination trials were performed to evaluate the effect of the fungal isolates in embryo growth and differentiation. The seeds from one mature capsule were superficially disinfected by immersion in ethanol 90% for one min, sodium hypochlorite 10% for two min and tree washes in sterile deionized water. The disinfected seeds were suspended in 30 mL sterile deionized water, and 300 µL of the suspension were dispersed in Petri dishes containing 25 mL of oatmeal agar (OMA plus streptomycin 100 mM). A fungal plug (ø 5 mm) was placed in the center of the plate containing OMA and incubated for a maximum of six months in darkness at 25 ± 1 °C. The effect of fungi on seed germination after 60 days was estimated, as reported in Vasudevan et al. [27] with modifications: stage 0, presence of embryo, without testa modification; stage 1, imbibed embryo, swollen, partially covered by testa; stage 2, enlarged seed without testa; stage 3, protocorms with rhizoids; stage 4, protocorms with pointed shoot apex and rhizoids; stage 5, emergence of first leaf. A germination index was calculated according to the formula proposed by Valadares et al. [23]:

$$\mathrm{GI}=\frac{N_{1}+N_{2}\times 2+N_{3}\times 3+N_{4}\times 4+N_{5}\times 5}{N_{0}+{\;N}_{1}+N_{2}+N_{3}+N_{4}+N_{5}}$$

where GI = germination index of 100 evaluated seeds and N_0_, N_1_, N_2_, N_3_, N_4_ and N_5_ are the numbers of seeds at stages 0, 1, 2, 3, 4 and 5, respectively. The most effective strain for seed germination was deposited to the Chilean Collection of Microbial Genetic Resources at Instituto de Investigaciones Agropecuarias (INIA), under the code 1030.

Quantitative data were analyzed by ANOVA. If the *p* value indicated significant differences between treatments (*p* < 0.05), post-hoc pair-wise comparisons were performed using the SD of means and Tukey’s multiple range test. Statistical significance was set at *p* < 0.05. All statistical tests were conducted using the *R* software (R Core Team 2018; https://www.R-project.org).

## 3. Results

During sampling, 13 terrestrial orchids belonging to the genera *Chloraea*, *Gavilea* and *Codonorchis* were found at the 11 different sampling sites considered for our analyses.

Mycorrhizal fungi were isolated from all orchid species at different frequencies, as shown in Table 2 and Table 3, and belonged to the order Cantharellales. The isolates FO1, FO2, FO3, FO4, FO5 and FO6 showed high similarity with different *Ceratobasidium* strains, as shown in Table 3, whereas the isolates FO7, FO8 and FO9 showed high similarity with diverse *Tulasnella* strains, as shown in Table 3. Regardless of the sampling site, *Ceratobasidium* spp. were the most frequent strains, accounting for approximately 90% of the isolates, especially in the orchids sampled near the coastal mountains. By contrast, orchids from the sites near the Andes mountains (i.e., *Chloraea alpina*, *Chloraea magellanica* and *Gavilea lutea*) showed less success in the isolation of mycorrhizal fungi, and the obtained isolates belonged mainly to *Tulasnella* spp.

**Figure 1:**
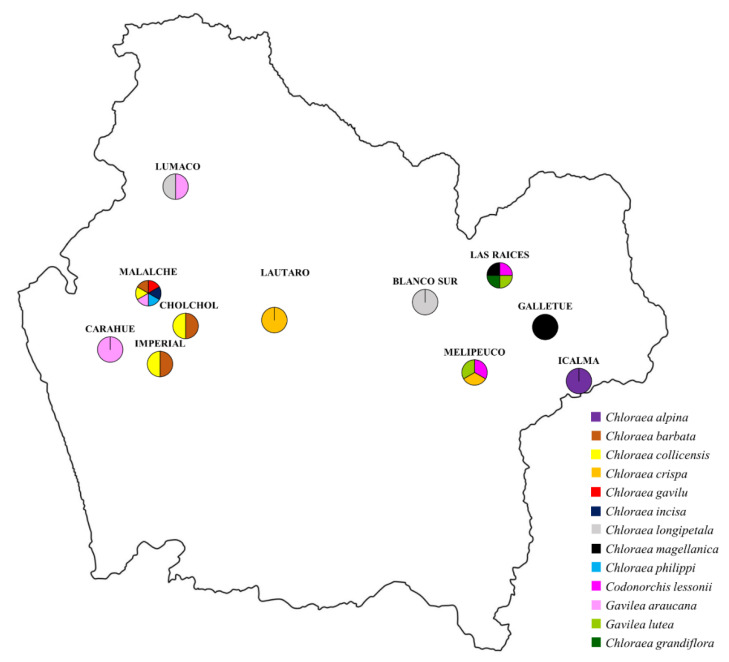
Sampling sites and presence of the analyzed orchid plants in Araucanía Region in southern Chile.

At the sites with orchid species living sympatrically, we found sites with different mycorrhizal fungi colonizing the roots (i.e., orchids from Malalche), as shown in Figure 1 and Table 3, and sites with conserved mycorrhizal fungus (i.e., orchids from Las Raices), as shown in Figure 1 and Table 3.

**Figure 2:**
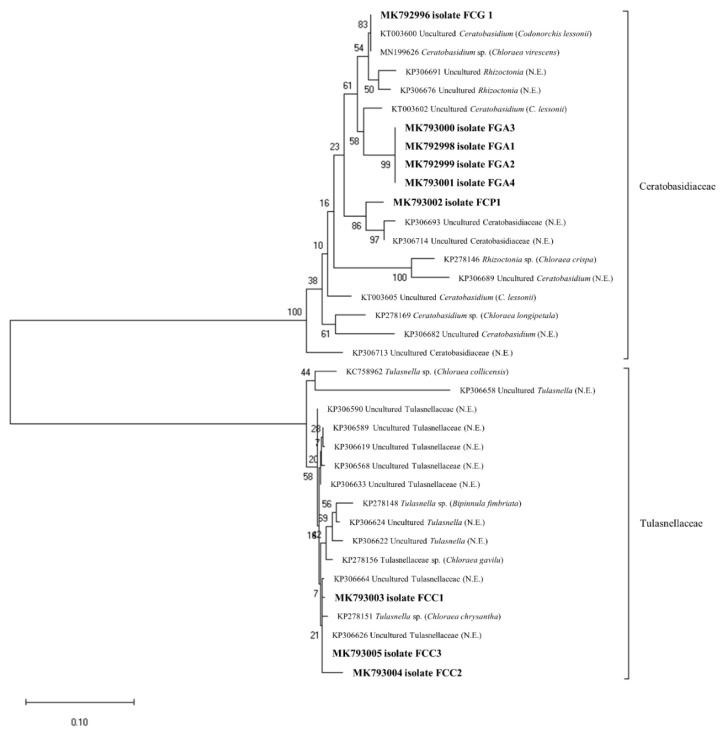
Maximum likelihood tree of ITS sequences of orchid mycorrhizal fungi isolated from orchid from Araucanía Region, Southern Chile (in bold). The tree also includes mycorrhizal fungi isolated from other Chilean native orchids.

For the phylogenetic analyses, we considered 178 sequences of mycorrhizal fungi isolated from native Chilean orchids available in the GenBank database (retrieved in October 2019). Such sequences were reduced to 27, based on the sequence similarity, and included in the phylogenetic analyses. The phylogenetic analyses showed that mycorrhizal fungi isolated from the orchid analyzed in this study belonged to four OTUs, three from Ceratobasidiaceae and one from Tulasnellaceae, as shown in Figure 2. In the Ceratobasidiaceae, the isolates FO2, FO3, FO4 and FO5 were assigned to OTU1, whereas the isolates FO6 and FO1 were assigned to OTU2 and OTU3, respectively. In the Tulasnellaceae, the isolates FO7, FO8 and FO9 were assigned to the OTU4.

To evaluate the effect of mycorrhizal fungi on seed germination, only a representative strain from each OTU was selected, as shown in Table 4. Almost 9 out of 12 orchids tested (except *Chloraea gavilu* which have no viable seeds) were germinated at different rates with almost one of the tested isolates, excluding the orchids *Codonorchis lessonii*, *Gavilea araucana* and *Chloraea incisa*, which had no effect on embryo growth, as shown in Table 4. The results showed that strain FO5 from OTU3 was the most effective isolate able to promote germination and differentiation, leading to the massive germination of *Chloraea crispa* seeds with protocorms up to stage 4 in some cases, as shown in Table 4. However, such germination seems to be species-specific, because some OTUs promote the germination of some species but fail in others, as shown in Table 4. The highest germination indexes were obtained in *Chloraea crispa* (GI = 3.26) and *Chloraea collicensis* (GI = 3.08). The symbiotic germination was significantly low in the *Tulasnella*-associated orchids, whereas the plants associated with *Ceratobasidium* strains had a higher germination index, as shown in Table 4. We also detected that, when protocorm development was complete, most of them failed to advance to the plantlet stage without changes in protocorm growth after three months of co-culture.

## 4. Discussion

Our study analyzes the presence of orchid mycorrhizal fungi in the roots of native terrestrial orchids growing in sites with a high density of orchid plants and tested their roles in seed germination. Despite mycorrhizal fungi associated with terrestrial orchids having been previously identified, such studies have reported a high diversity of OMF associated with the target plants, including mycorrhizal and non-mycorrhizal fungal endophytes, which agrees with the results obtained in our study [19,22,32]. Such diversity is influenced by the high diversity of forests present in the sampling sites, which certainly influences the symbiosis between orchids and fungi. Additionally, several exogenous mechanisms related to the ecosystem can influence the composition of endophytes, including the mycoheterotrophic strategy of the plant (autotrophic, fully or partially mycoheterotrophic), nutrient availability and surrounding plant species [33,34]. Similar to our study, Fracchia et al. [35] analyzed the potential of mycorrhizal fungi isolated from the terrestrial Andean orchids, *Chloraea riojana* and *Aa achalensis,* for promoting seed germination in threatened species. Our results showed that some of the *Ceratobasidium* isolates (especially from orchids sampled in coastal area grassland) can positively induce seed germination to different degrees despite the fact that the transition from protocorm to plantlet was difficult. Such mechanisms may reflect the need for associations with other microorganisms, which can influence the transition from protocorm to plantlet under field conditions, similar to what is reported for the terrestrial orchids *Goodyera pubescens* and *Paphiopedilum appletonianum* [11,36]. A previous study analyzing root fungal endophytes on native terrestrial orchids from south–central Chile have isolated and identified several fungal endophytes from Chilean orchids, including non-mycorrhizal fungi with roles in plant growth promotion, such as *Chaetomium* spp., *Phialocephala* spp. and *Leptodontidium* spp. [37,38]. Additionally, bacterial endophytes may positively interact to improve the nutrition and growth of mycoheterotrophic species, such as in the case of *Arachnitis uniflora* and *Dendrobium catenatum* [39,40]. Associations with such microorganisms are necessary for orchids, especially after the initial mycoheterotrophic stage, for them to become established in the ecosystem [39,41]. Our results showed that, after two months of symbiotic germination, the growing protocorms slow their growth, probably by the lack of appropriated soil-borne fungi able to contribute synergistically to plant growth. This diverse class of fungi can also be isolated from mycorrhizal tissues and can have positive roles in the orchid life cycle [9,42,43].

Despite the fact that asymbiotic seed germination has been tested in Andean terrestrial orchids with positive results, such processes are far from what occurs under natural conditions and the plants lack mycorrhizal benefits, such as nutrition, stress tolerance and defense against plant pathogens [44,45,46,47]. Our results agree with resent studies into mycorrhizal fungi associated with native orchids from Chile, which have shown contrasting abilities to induce seed germination under laboratory conditions, which denotes the crucial role of the OMF in providing the initial C to start germination and promote embryo growth and differentiation [19,22]. Hence, the use of mycorrhizal fungi in seed germination strategies is a crucial step to improve plant performance under field conditions. It would be very interesting to characterize other mycorrhizal fungi able to associate with germinated protocorms in the field, in order to better understand a crucial symbiotic step that will help improve the survival rates of plantlets derived from in vitro symbiotic germination assays.

Tulasnellaceae have been described as common mycorrhizal fungi associated with photosynthetic terrestrial orchids [33,48]. Such associations are conserved in terrestrial orchids from the southern Andes, where several *Rhizoctonia*-like strains can be isolated from native orchids [20,22,49]. Additionally, the phylogenetic analyses showed that some of the isolates have high similarity with other mycorrhizal fungi isolated from the orchids *C. lessonii* and *Chloraea chrysantha*, among others, as shown in Figure 2. Unlike our previous study, conducted in south–central Chile, where we identified *Tulasnella* spp. as the main mycorrhizal fungi associated with terrestrial orchids from the Maule region [19], in this study we identified *Ceratobasidium* spp. as the most frequent isolates and some strains were effective at inducing the seed germination of the related orchids species. Similar results were reported in the orchids *Bipinnula volckmannii* and *Bipinnula apinnula,* despite the fact that mycorrhizal fungi isolated from adult roots (*Ceratobasidium* spp.) mostly fail to induce embryo growth and differentiation, suggesting that mycorrhizal fungi isolated from adult plants may not be as effective at inducing the germination and development of the protocorms as mycorrhizal fungi isolated from protocorms, such as was the case in the results of our study in orchids from the sampling sites near the Andean mountains [21,50]. Furthermore, temporal variations in mycorrhizal fungi must to be considered by the effect of the habitat conditions as well as neighboring plant species during the vegetative or reproductive growth of some terrestrial orchids [51,52], a process that has certainly influenced the presence of fungi in the orchid roots analyzed in our study.

Symbiotic seed germination in orchids is a complex process, particularly in terrestrial orchids, where different factors affect germination success. Our results did not show a conserved mycorrhizal fungus capable of promoting embryo growth and differentiation, suggesting specific fungal partners for each orchid species, which is consistent with Meng et al. [50], who showed that mycorrhizal fungi isolated from advanced seedlings are more efficient at inducing seed germination than fungi isolated from adult mycorrhizal roots of the terrestrial orchid *Arundina graminifolia*, a process that may explain the low germination rates obtained in our studies, especially in the plants from the Andean sites.

Mycorrhizal fungi associated with terrestrial orchids have demonstrated a direct influence on the distribution of orchid populations. McCormick et al. [53] reported that the dependence of orchids on mycorrhizal fungi can affect the distribution range of orchid species and it is necessary to know which orchids need particular mycorrhizal fungi for germination and plantlet development and whether there is an influence from other fungal endophytes in plantlet development. Such variable dependence on fungi may reflect a dynamism in the association of orchids with fungi at different plant stages (i.e., protocorm plantlet and adulthood) and certainly influences the presence of orchid plants at sites with abundant populations. Hence, it is crucial to know the mycorrhizal fungi associated with native orchids in specific microhabitats to develop safeguarding strategies for endemic orchids under imminent threat [8].

## 5. Conclusions

In this study we isolated and characterized mycorrhizal fungi associated with thirteen terrestrial orchids from native ecosystems in the region of La Araucanía, southern Chile, which has expanded the knowledge of mycorrhizal associations with native orchids in Chile. We detected that mycorrhizal fungi belonging to Ceratobasidiaceae were the most frequently isolated and effective at promoting seed germination to differing degrees. Such isolated microorganisms can be stored and implemented in germination programs of threatened species involving compatible mycorrhizal fungi.

## Figures and Tables

**Table 1:** List of plant and locations of orchids sampled in the experiments.

Species	Location	Sample Site	Number of Root Samples
*Chloraea alpina*	Icalma (38°46′36.4″ S 71°09′44.5″ W)	Grassland	4
*Chloraea barbata*	Imperial (38°43′31.5″ S 72°59′45.0″ W) Cholchol (38°36′40.9″ S 72°49′16.0″ W) Malalche (38°34′53.0″ S 72°56′01.8″ W)	Grassland Grassland Grassland	4 4 4
*Chloraea collicensis*	Imperial (38°43′31.5″ S 72°59′45.0″ W) Cholchol (38°36′40.9″ S 72°49′16.0″ W) Malalche (38°34′53.0″ S 72°56′01.8″ W)	Grassland Grassland Grassland	4 4 4
*Chloraea crispa*	Melipeuco (38°50′12.2″ S 71°39′36.8″ W) Lautaro (38°35′15.1″ S 72°26′52.1″ W)	Wayside Rock	4 4
*Chloraea gavilu*	Malalche (38°34′01.7″ S 72°56′57.3″ W)	Understory	4
*Chloraea grandiflora*	Las raices (38°27′34.7″ S 71°30′09.1″ W)	Understory	2
*Chloraea incisa*	Malalche (38°34′01.1″ S 72°57′21.7″ W)	Understory	1
*Chloraea longipetala*	Blanco sur (38°30′40.5″ S 71°51′01.5″ W) Lumaco (38°10′06.2″ S 72°51′42.2″ W)	Wayside Wayside	2 1
*Chloraea magellanica*	Las raices (38°27′34.7″ S 71°30′09.1″ W) Galletue (38°37′05.8″ S 71°26′02.4″ W)	Grassland Understory	3 3
*Chloraea philippi*	Malalche (38°34′05.5″ S 72°57′17.2″ W)	Understory	2
*Codonorchis lessonii*	Melipeuco (38°45′02.8″ S 71°36′09.8″ W) Las Raíces (38°27′33.5″ S 71°30′35.4″ W)	Understory Understory	2 2
*Gavilea araucana*	Malalche (38°33′38.5″ S 72°56′19.5″ W) Carahue (38°41′32.2″ S 73°10′23.8″ W) Lumaco (38°10′06.2″ S 72°51′42.2″ W)	Understory Wayside Wayside	2 1 4
*Gavilea lutea*	Las Raíces (38°27′32.6″ S 71°30′26.3″ W) Melipeuco (38°45′02.8″ S 71°36′09.8″ W)	Understory Understory	3 2

**Table 2:** Occurrence and morphological characteristics of the root fungal isolates associated to terrestrial orchids from the region of La Araucanía, Southern Chile.

Isolate	Number of Strains	Isolation Frequency	Growth Rate (m day^−1^)	Color
FO1	6	0.08	7.5 ± 2.2	White/cream
FO2	17	0.24	6.2 ± 1.5	White
FO3	11	0.15	6.8 ± 3.0	White
FO4	6	0.08	5.3 ± 0.4	White
FO5	8	0.11	6.0 ± 1.4	White
FO6	13	0.18	5.7 ± 0.6	White
FO7	4	0.06	3.5 ± 0.2	Light brown
FO8	5	0.07	2.8 ± 0.1	Light brown
FO9	2	0.03	4.1 ± 0.4	Light brown

**Table 3:** Molecular identification of culturable mycorrhizal fungi isolated from peloton-containing roots, based on the closest match in the GenBank database.

Fungal Isolate	GenBank Accession Number	Isolation Source	Close Relatives (Accession Number)	Identity (%)	Source	Reference
FO1	MK792996	*Gavilea araucana, Chloraea gavilu*	*Ceratobasidium* sp. FN812725	99	Air	Jurado et al. [28]
FO2	MK792998	*G. araucana, Chloraea longipetala, Chloraea barbata, Chloraea collicensis*	Uncultured Ceratobasidiaceae JQ972130	100	Orchid root	Pandey et al. [29]
FO3	MK792999	*Chloraea philippi, Chloraea crispa, C. barbata*	Uncultured Ceratobasidiaceae JQ972130	100	Orchid root	Pandey et al. [29]
FO4	MK793000	*Chloraea collicensis*	Uncultured Ceratobasidiaceae JQ972129	100	Orchid root	Pandey et al. [29]
FO5	MK793001	*C. crispa, C. longipetala*	Uncultured Ceratobasidiaceae JQ972130	100	Orchid root	Pandey et al. [29]
FO6	MK793002	*C. philippi, G. araucana, Chloraea incisa*	Uncultured Ceratobasidiaceae FJ788720	97	Orchid mycorrhizal root section	Waterman et al. [30]
FO7	MK793003	*C. collicensis*	Uncultured Tulasnellaceae JF691471	99	Orchid root	Martos et al. [31]
FO8	MK793004	*Gavilea lutea, Chloraea alpina, Codonorchis lessonii*	*Tulasnella* sp. KP278150	98	*Chloraea gavilu*	Herrera et al. [19]
FO9	MK793005	*Chloraea grandiflora, Chloraea magellanica*	*Tulasnella* sp. KJ713701	100	*Gavilea australis*	Fracchia et al. [32]

**Table 4:** Symbiotic germination index (GI) for the four tested isolated fungal strains on twelve orchid seeds over 60 days of in vitro culture. Results are means ± standard deviation for *n* = 5. Differrent letters in the GI of each OTU are significantly different according to Tukey’s multiple range test (*p* < 0.05).

Orchid Species	Control	OTU1 *Ceratobasidium* sp.	OTU2 *Ceratobasidium* sp.	OTU3 *Ceratobasidium* sp.	OTU4 *Tulasnella* sp.
*Chloraea alpine*	0	0.02 ± 0.0 ^c^	0.07 ± 0.0 ^d^	0	1.76 ± 0.2 ^a^
*Chloraea magellanica*	0	0	0.05 ± 0.0 ^d^	0	0.07 ± 0.0 ^cd^
*Chloraea grandiflora*	0	0.03 ± 0.0 ^c^	0.05 ± 0.0 ^d^	0.02 ± 0.0 ^d^	0
*Gavilea lutea*	0	0	0	0	1.27 ± 0.3 ^b^
*Codonorchis lessonii*	0	0	0	0	0
*Gavilea araucana*	0	0	0	0	0
*Chloraea longipetala*	0	0.05 ± 0.0 ^c^	0.12 ± 0.0 ^c^	1.83 ± 0.3 ^c^	0
*Chloraea barbata*	0	1.67 ± 0.3 ^a^	1.96 ± 0.0 ^b^	2.71 ± 0.2 ^b^	0.26 ± 0.1 ^c^
*Chloraea collicensis*	0	1.72 ± 0.2 ^a^	1.84 ± 0.2 ^b^	3.08 ± 0.2 ^a^	0.07 ± 0.0 ^cd^
*Chloraea crispa*	0.04 ± 0.0 ^ns^	1.97 ± 0.4 ^a^	2.39 ± 0.2 ^a^	3.26 ± 0.1 ^a^	1.17 ± 0.2 ^b^
*Chloraea incisa*	0.0	0	0	0	0
*Chloraea philippi*	0.05 ± 0.0 ^ns^	0.54 ± 0.0 ^b^	0.28 ± 0.1 ^c^	0.04 ± 0.0 ^d^	0.03 ± 0.0 ^d^

^ns^ Non-significant.
